# Health care providers’ weight management practices for adolescent obesity and alignment with clinical practice guidelines: a multi-centre, qualitative study

**DOI:** 10.1186/s12913-020-05702-8

**Published:** 2020-09-10

**Authors:** M. Kebbe, A. Perez, A. Buchholz, S. D. Scott, T.-L. F. McHugh, M. P. Dyson, G.D.C. Ball

**Affiliations:** 1 Department of Pediatrics, Faculty of Medicine & Dentistry, Edmonton Clinic Health Academy, University of Alberta, 11405 – 87 Avenue, Edmonton, Alberta T6G 1C9 Canada; 2 Centre for Healthy Active Living, Children’s Hospital of Eastern Ontario, Ottawa, Ontario Canada; 3 Faculty of Nursing, University of Alberta, Edmonton, Alberta Canada; 4 Faculty of Kinesiology, Sport, and Recreation, University of Alberta, Edmonton, Alberta Canada

**Keywords:** Adolescent, Delivery of health care, Lifestyle, Obesity management

## Abstract

**Background:**

Clinical practice guidelines (CPGs) include evidence-based recommendations for managing obesity in adolescents. However, information on how health care providers (HCPs) implement these recommendations in day-to-day practice is limited. Our objectives were to explore how HCPs deliver weight management health services to adolescents with obesity and describe the extent to which their reported practices align with recent CPGs for managing pediatric obesity.

**Methods:**

From July 2017 to January 2018, we conducted a qualitative study that used purposeful sampling to recruit HCPs with experience in adolescent weight management from multidisciplinary, pediatric weight management clinics in Edmonton and Ottawa, Canada. Data were collected using audio-recorded focus groups (4–6 participants/group; 60–90 min in length). We applied inductive, semantic thematic analysis and the congruent methodological approach to analyze our data, which included transcripts, field notes, and memos. Qualitative data were compared to recent CPGs for pediatric obesity that were published by the Endocrine Society in 2017. Of the 12 obesity ‘treatment-related’ recommendations, four were directly relevant to the current study.

**Results:**

Data were collected through three focus groups with 16 HCPs (*n* = 10 Edmonton; *n* = 6 Ottawa; 94% female; 100% Caucasian), including dietitians, exercise specialists, nurses, pediatricians, psychologists, and social workers. We identified three main themes that we later compared with CPG recommendations, including: *(i) discuss realistic expectations regarding weight management* (e.g.*,* shift focus from weight to health; explore family cohesiveness; foster delayed vs instant gratification), *(ii) personalize weight management* (e.g.*,* address personal barriers to change; consider developmental readiness), and *(iii) exhibit non-biased attitudes and practices* (e.g.*,* de-emphasize individual causes of obesity; avoid making assumptions about lifestyle behaviors based on weight). Based on these qualitative findings, HCPs applied all four CPG recommendations in their practices.

**Conclusions:**

HCPs provided practical insights into *what* and *how* they delivered weight management for adolescents, which included operationalizing relevant CPG recommendations in their practices.

## Background

Adolescent obesity is concerning given its high prevalence [1] and associations with adverse physical, social, and emotional health consequences in adolescence and later in life [2–4]. It also poses a considerable financial burden on the health care system, including direct medical costs and indirect productivity losses [5]. These costs persist into adulthood since ~ 70% of adolescents with obesity become adults with obesity [6]. These data provide strong justification for developing and implementing effective interventions to manage obesity in adolescents in order to reduce morbidity, mortality, and costs associated with obesity in adulthood.

Managing adolescent obesity is a challenging task that health care providers (HCPs) are well-suited to address. HCPs seek to provide high quality care for adolescents enrolled in weight management and are valued sources of health information and expertise. HCPs who practice in multidisciplinary teams often have the ability to deliver intensive family-based weight management interventions, including frequent office visits and extended follow-up [7]. In the short-term, multidisciplinary, family-based behavioral interventions can be effective (albeit modestly) for managing obesity in adolescents [8]. The successful management of obesity depends largely on the extent to which healthy lifestyle behavior changes are adopted and maintained. Based on a recent review [9], adolescents experience a number of challenges in adopting healthy lifestyle habits. Of these challenges, some require higher organizational and policy changes (e.g.*,* food advertisements that target adolescents) compared with intra- and inter-individual level factors (e.g.*,* addressing mental health concerns) that may be more amenable to change by HCPs and families.

To our knowledge, despite HCPs’ front-line experiences in supporting adolescents with obesity, their key practices have not been described extensively in the literature nor the extent to which these align with recommendations from existing clinical practice guidelines (CPGs) [10]. Typically, pediatric obesity CPGs provide recommendations for growth monitoring as well as prevention and management strategies. While they may offer HCPs with practice-based recommendations for children, adolescents, and their families, they often lack specificity about *how* recommendations should be operationalized. This may result in inconsistencies in how recommendations are implemented across practice settings for weight management, and how best to implement them based on evidence for optimal care. The objectives of this study were to explore how HCPs deliver weight management health services to adolescents with obesity (and their parents) and describe the extent to which their reported practices align with recent CPGs for managing pediatric obesity.

## Method

### Study design

Conducted from July 2017 to January 2018, our multi-centre, qualitative study was informed by the qualitative description (QD) method [11]. QD is well-suited to behavioral research as it aims to provide a comprehensive summary of events in the everyday terms of those events. For our purposes, QD provided a straightforward conceptual summary of how HCPs deliver weight management health services to adolescents with obesity based on the manifest content of the data collected without requiring detailed interpretive interference [11]. We received research ethics and operational approvals from the University of Alberta (Edmonton, AB), Alberta Health Services (Edmonton, AB), and Children’s Hospital of Eastern Ontario (Ottawa, ON) prior to study commencement. We adhered to the Consolidated criteria for reporting qualitative studies (Additional file 1).

### Participants and recruitment

Using purposeful sampling, we recruited HCPs from two pediatric weight management clinics (Pediatric Centre for Weight and Health [PCWH], Stollery Children’s Hospital, Edmonton, AB; Centre for Healthy Active Living [CHAL], Children’s Hospital of Eastern Ontario, Ottawa, ON). To be included in our study, HCPs (e.g.*,* dietitians, mental health professionals, nurses, pediatricians) had to have ≥6 months of experience in adolescent weight management to ensure that they had gained clinical insight and experience working with that age group. We scheduled focus groups with HCPs at each site and obtained written, informed consent prior to each focus group. All HCPs were offered a $25 Amazon gift card as a token of appreciation for study participation.

### Data collection

We held three semi-structured, in-person focus groups (60–90 min in duration; two in Edmonton [including one pilot] and one in Ottawa). The focus group interview guide was developed by MK, informed through discussions with team members who have content (obesity) and methodological (qualitative inquiry) expertise (AP, TLFM, SDS, GDCB), and refined iteratively (Additional file 2). We asked HCPs (4–6 per focus group) open-ended questions regarding their experiences in delivering weight management to adolescents. Probing questions and prompts were used to encourage participants to provide more in-depth responses to the questions. Following the pilot interview, minor editorial changes and refinements were made to improve clarity, readability, and sequencing of the interview questions. MK and AP, both of whom are formally trained in qualitative inquiry, moderated the focus groups, debriefed afterwards, and documented field notes and memos. Focus groups were audio-recorded, uploaded to an online and secure file sharing platform called *LabKey* (Women and Children’s Health Research Institute, University of Alberta), and transcribed verbatim (Translation Agency of Alberta). At the completion of each focus group, we collected descriptive and demographic information from HCPs using a password-protected survey that was disseminated via email.

### Data analysis

As a first step, a team member (MK) verified the de-identified transcripts line-by-line against the audio recordings for completeness and accuracy, which were imported to *NVivo 11* (QSR, Melbourne, Australia). Data were analyzed independently and iteratively (by MK and AP); we did not observe appreciable differences across clinics regarding HCP practices, so all three focus groups (including the pilot) were combined for analysis. Both team members followed processes described by Braun and Clarke [12] for thematic analysis, including reading and re-reading the transcripts for data familiarization, developing a coding scheme (organized by topic, root codes, and code names), identifying exemplar quotes, and having team discussions (MK, AP, GDCB). We also employed Duggleby’s congruent methodological approach to analyze group interaction data and non-verbal interchanges among participants separately from individual or group data using the same methodological approach [13]. This entailed situating the patterns or themes identified across the focus groups within group interactions from field notes, including the frequency and intensity of expressed themes, areas of agreement and disagreement, contradictions in the discussion, and viewpoints that may have been silenced or alliances formed. Since HCPs shared similar descriptive characteristics, we chose to remove identifiers (e.g.*,* profession) from the provided quotes to ensure anonymity.

Following data collection and thematic coding, themes were compared with recommendations from recent CPGs [10]; HCPs were naïve to this activity during the focus groups. We chose to make comparisons in relation to the most up-to-date international CPGs for managing pediatric obesity, which were published in 2017 by the Endocrine Society [10]. Recommendations that were deemed to be unrelated (e.g.*,* strategies for dietary and physical activity changes) or irrelevant (e.g.*,* neither site currently offered bariatric surgery as a treatment option) to HCP practices at the study sites were excluded. None of the recommendations were specific to adolescents (i.e.*,* included the full pediatric age range). Recommendations relevant to how HCPs should practice pediatric weight management (a total of four out of 12) were reviewed and retrieved, then compared and contrasted with the themes generated from the qualitative data collected from HCPs. HCP practices were deemed to align with CPG recommendations if they matched in content. Any discrepancies throughout the data analysis process (qualitative analysis and CPG compare/contract activity) were resolved through discussion, which resulted in achieving consensus (between MK, AP, GDCB).

### Methodological rigor

We used several strategies to ensure methodological rigor, including investigator responsiveness, methodological coherence, sampling adequacy, and theoretical thinking [14]. MK also examined her own role as a researcher through an ongoing critical reflection, including personal, functional, and disciplinary reflexivity [15], and how these characteristics may have shaped the research process and influenced data collection and analysis. Further, the recommendations were reviewed and retrieved by one team member (MK) and verified for accuracy and completeness by another (GDCB).

## Results

A total of 16 HCPs (mean [standard deviation, SD] age: 43.1 ± 10.4 years old) participated in our study, most of whom were female (*n* = 15; 94.0%), Caucasian (*n* = 16; 100.0%), and had 0.5 to 15 years (mean [SD]: 5.3 ± 4.7 years) of experience providing weight management services to adolescents with obesity. Findings were represented by three themes that summarized HCPs’ approaches to delivering weight management support to adolescents with obesity, including: *(i)* discuss realistic expectations regarding weight management, *(ii)* personalize weight management, and *(iii)* exhibit non-biased attitudes and practices. HCPs applied, and expanded beyond, all four CPG recommendations in their practices, described below and in Table 1.

**Table 1:** Recommendations from clinical practice guidelines compared and contrasted with HCP practices

CPG Recommendations	HCP Practices	Theme
4.1 We recommend that clinicians prescribe and support intensive, age-appropriate, culturally sensitive, family-centered lifestyle modifications (dietary, physical activity, behavioral) to promote a decrease in BMI. (1| ⊕ ⊕ ⊕ O)4.1	HCPs prescribed and supported age-appropriate, culturally sensitive, family-centered lifestyle modifications.	*Personalize weight management*
	HCPs encouraged adolescents to adopt healthy lifestyle behaviors irrespective of weight loss and defined ‘success’ based on improved health.	*Discuss realistic expectations regarding weight management*
4.5 We suggest that the health care team identify maladaptive rearing patterns related to diet and activity and educate families about healthy food and exercise habits. (2| ⊕ OOO)	HCPs probed for and addressed individual, interpersonal, and environmental issues (if applicable) experienced by parents and adolescents, including lifestyle practices related to diet and activity.	*Personalize weight management*
4.6 We suggest that the health care team probe for and diagnose unhealthy intrafamily communication patterns and support rearing patterns that seek to enhance the child’s or adolescent’s self-esteem. (2| ⊕ OOO)4.6	HCPs addressed unhealthy communication patterns with families to clarify expectations of each member in their weight management efforts.	*Discuss realistic expectations regarding weight management*
	HCPs promoted positive self-image and acceptance in adolescents directly to help alleviate weight bias concerns and reduce stigma.	*Exhibit non-biased attitudes and practices*
4.7 We suggest that the health care team evaluate for psychosocial comorbidities and prescribe assessment and counseling when psychosocial problems are suspected. (2| ⊕ OOO)	HCPs probed for and addressed individual and interpersonal issues experienced by parents and adolescents, including psychosocial concerns.	*Personalize weight management*
–	HCPs had discussions with adolescents and parents about treatment expectations and timelines at the point when they enrolled in weight management as well as during their course of treatment.	*Discuss realistic expectations regarding weight management*

*Strong recommendations use the phrase “we recommend” and the number 1. Weak recommendations use the phrase “we suggest” and the number 2. Cross-filled circles indicate the quality of the evidence, such that ⊕ OOO denotes very low quality evidence and ⊕ ⊕ ⊕ O, moderate quality*

### HCP practices in adolescent weight management

#### Discuss realistic expectations regarding weight management

HCPs shared that adolescents and their parents may attend initial clinical appointments with misunderstandings or misperceptions about the health services available to them, which can lead to unrealistic treatment expectations. For instance, HCPs stated that weight loss was the focus of most families, but all HCPs agreed that they prioritized health over weight in their practices. This was illustrated in the following quote: *“We are willing to help people develop healthy behaviors and actually decrease weight [loss] gratification. So, that’s why we’re not always having that conversation [weight loss] necessarily and it’s tricky because you kind of want to validate and acknowledge how they are feeling about their weight and their bodies, but at the same time, you don’t want to be over-focusing on it.”*

HCPs highlighted the importance of facilitating concordance between adolescents’ and parents’ treatment expectations. Some described how parents tended to hold adolescents to high standards for weight management regardless of their own participation in making lifestyle behavior changes. According to HCPs, these high expectations may have stemmed from parental perceptions of adolescents’ increasing independence and distancing from family to spend more time with peers and friends. As one HCP stated: *“She [patient] is embedded in a fairly strong family system in some ways [although], once again, this family is still pointing the finger at her and saying you’re still the one that has to really make the change [due to difficulties in changing deeply rooted parental lifestyle habits].”*

HCPs emphasized that they often worked with families to help them form realistic expectations and timelines related to changes in adolescents’ health outcomes. As one HCP mentioned: *“I think […] people are looking for weight loss, and dramatic [speed and weight changes] […] The idea of the biggest loser, so when [adolescents and parents] come in and they don’t see this dramatic weight loss change, then they surrender any kind of effort that they’re already doing. So kind of what is a person’s expectations, what is it that they want, what is their own goal in terms of their personal health, and I think that that’s part of the conversation that maybe has to happen a little bit more.”* HCPs described how common it was for adolescents to have unrealistic expectations in other areas of life, which carry over to expectations for weight management and behavior change. For example, as one HCP stated: *“It’s a little bit challenging, and it feeds into where our culture is at. I’d say kind of teen generation, is where they are getting information so quickly and things are coming at them and they are processing things very quickly. The perception of taking time to change or process a body of information or make long term decisions is off the radar.”*

#### Personalize weight management

HCPs shared that families often faced a number of barriers that hampered their ability to effectively manage adolescents’ weight, including individual (e.g.*,* low readiness for change, high use of technology), interpersonal (e.g.*,* peer pressure from adolescents’ social network, poor family mental health), and environmental (e.g.*,* accessibility to inexpensive junk food and sugar-sweetened beverages) factors and the need to address these barriers, if applicable, for effective weight management. While some HCPs reported that low self-esteem and confidence hampered adolescents’ ability to make healthy lifestyle behavior changes, others commented that a lack of parental engagement was a critical issue. HCPs added that while some parents held adolescents accountable because they perceived their high level of autonomy, other parents were not supportive for individual psychological reasons: *“It’s not a willingness to place ownership on a teen, there’s a big barrier to families not being able to provide that support for their own emotional health reasons […] So that doesn’t make it easier for a teen to make that change, right, when the family can’t help them the way that they would want to be helped.”* In response, HCPs tailored their practices to reflect differences within and between families.

HCPs were unanimous in endorsing attempts to change lifestyle behaviors earlier to optimize the likelihood of success: *“I find the younger [the adolescent for whom] the parents can structure this [gradual lifestyle changes], the more success they have. So, if we’re talking 13, they might have a bit more success with that [lifestyle change] than when the kids are 17 and a half.”* In addition to cultural considerations, another HCP shared how she tailors her counseling based on adolescents’ age and developmental capacity: *“It [expectations for change] depends on their developmental growth profile, their cognitive ability, where they are at. Like a thirteen-year-old will understand it very differently from a sixteen-year-old […] So I use different wording to illustrate that, but for the sixteen-year-old, most sixteen-year-olds that are cognitively kind of in a normal range, I get the sense that they say that there is this light bulb that goes off: ‘Oh, okay, this isn’t going to make me into like Mr. Muscle by next week’.”*

#### Exhibit non-biased attitudes and practices

HCPs suggested that societal and social pressures around health and weight may have influenced how adolescents with obesity perceived themselves; that is, a perception of self that is not ‘normal’. HCPs described that *(i)“People feel guilty about themselves, but this [the individual adolescent] isn’t down to be questioned, but the society.”* and that within societies, *(ii) “There is also the social pressures whether it’s from your friends or whoever they see on social media to look a certain way, be a certain way, and act a certain way.”* In relation to social acceptability, one HCP said: *“I think one of the things that makes it hard for teens to make those changes is they want to be doing what everybody else is doing, so they don’t want to be seen as the person who’s having the healthy meal at lunchtime. You don’t know how many times I get a kid say ‘Well, everybody else is doing it, everybody else takes their phone to bed, everybody else is answering, I’m the only kid in the school that doesn’t do that’.”* To this extent, HCPs’ practices included recognizing the similarities in lifestyle habits that exist between normal-weight adolescents and adolescents with obesity. As one HCP commented, to which others agreed: *“When you look at the lifestyle of our teens, it’s not that much of a different lifestyle of teens who don’t have obesity. So, they have very similar lifestyles as their peers and we’re asking them to change their lifestyle, to sort of go against, the bit of a norm out there […] If the messages were more universal, that would be really helpful for our kids and families.”*

HCPs strongly supported the idea that healthy bodies can come in different shapes and sizes. When asked about their perceptions, one HCP shared: “*I can’t disagree enough with that position [negatively viewing the normalization of obesity] and I’ve heard it many times. If you are looking to motivate an individual, you need to work on making someone feel good and accepted and safe. Taking that position is creating an environment where it is far from that and that, in my mind, has perpetuated our weight management issues in North America. That type of thinking and policy that has no evidence. If anything, the science contradicts that completely and it’s upsetting to me. It really does harm. It does harm. It’s done more harm to our kids and families. It’s got to stop.*” HCPs noted that adolescents may worry about being judged when they first attended the weight management centre: “*They don’t want to say too much, that’s what I always feel like. They are just worried whether because they’ve heard it at home, from school, or other physicians, that they are going to get judged by what their answers are, so the beginning might shape their answers a little bit differently.*” HCPs reported working as a multidisciplinary team to create a non-judgmental clinical environment and promote positive self-image and acceptance, although they believed these efforts were not always successful. As one HCP stated: “*I think they [adolescents] learn that it’s a safe place to be, that’s my sense. That anxiety that is there initially gets lessened; except when it doesn’t […] We’ve had very few drop out [weight management services] as a result of not feeling safe or feeling judged.”*

### HCP practices compared with CPGs

HCP practices and CPG recommendations spanned all three themes outlined above. Based on our qualitative findings, HCPs applied all four CPG recommendations in their practices, which included issues related to personalizing care based on age, culture, and family; encouraging healthy lifestyle behaviors for improved health; and addressing varied individual, interpersonal, and environmental factors, including unhealthy communication patterns and poor self-image to reduce weight bias and stigma. Absent from CPGs, HCPs in our study further emphasized the importance of defining ‘success’ based on improved health (as opposed to weight loss) and clarifying treatment expectations and timelines with adolescents and their parents.

## Discussion

In this qualitative study, HCPs described how they drew on their values, beliefs, and experiences in delivering multidisciplinary health services for weight management in adolescents. These included discussing realistic expectations regarding weight management, personalizing weight management, and exhibiting non-biased attitudes and practices. HCPs’ perspectives highlighted how they worked with adolescents with obesity and their parents, extending beyond the lifestyle and behavioral changes necessary to improve health and weight outcomes to include patient- and family-centred approaches to the education, counseling, and support they offered. HCPs identified practices that were consistent with CPG recommendations on the management of pediatric obesity, and offered additional, complementary approaches to help adolescents in weigh management.

All HCPs indicated weight loss to be a primary goal and expectation by families seeking weight management services. Body mass index reduction is in line with CPGs and expert recommendations encouraging patients to reach their *best weight*; that is, a weight that can be achieved while living the healthiest lifestyle that they can enjoy [10, 16]. However, adolescents often form unrealistic weight loss expectations, which can influence how they perceive success. For example, in a recent study, adolescents expected to achieve a median weight loss of 50 pounds and those who had higher expectations were more likely to discontinue weight management prematurely compared to their peers with lower expectations [17]. Given these unrealistic expectations and the well-documented challenges of losing and maintaining weight loss over time [18], it may be productive to shift families’ attention from a singular focus on weight loss to emphasize lifestyle and behavioral changes that can improve health outcomes and minimize the risk of obesity-related comorbidities [19]. This type of health-centred approach was practiced by HCPs in our study who reported encouraging adolescents to adopt healthy lifestyle behaviors irrespective of weight loss and defined ‘success’ based on improved health. In encouraging adoption of healthy lifestyle habits, HCPs emphasized the importance of having discussions with adolescents and parents about treatment expectations at the point when they enroll in weight management as well as during their course of treatment. It may also be valuable for HCPs to share information with families regarding the complex etiology of obesity [18, 20]; conceptualizing obesity as a complex condition that is linked with a range of factors beyond adolescents’ control (e.g.*,* restaurant portion sizes, school nutrition policies, transportation) may help families to identify and address the factors they do or do not have direct influence over. This is particularly important since some parents may hold their adolescents responsible for their excess weight as a way to lessen personal feelings of guilt or shame. In a previous report by our team, HCPs described using a strengths-based approach in delivering weight management services to adolescents; this included components of active listening and reflection, and was sometimes strengthened by using specific tools or strategies to engage adolescents [21]. Altogether, these approaches may help HCPs to align expectations with probable treatment outcomes, which might help to extend treatment duration and reduce attrition [22].

Our findings are consistent with CPGs that recommend HCPs to prescribe and support age-appropriate, culturally sensitive, family-centered lifestyle modifications [10]. Given that family-centered care includes the needs of individual family members, this approach to care has the potential to improve adolescent and family outcomes, experiences, and satisfaction with care [23]. However, the evidence for the effectiveness of parent-adolescent interventions remains limited [24]. Compared with literature that supports a role for parents in achieving successful outcomes in obesity interventions for children [25], there is limited evidence regarding parent-only and parent-adolescent interventions for managing obesity; existing studies tend to group children and adolescents together [24, 26], which fails to clarify the role that parents should play to optimize treatment outcomes for adolescents. For example, trial data from 8 to 14-year-olds support parent-only interventions as a viable and effective alternative to family-based treatment [26]. Yet, it is common for adolescents, especially those who have the developmental capacity for and interest in being involved in independent decision-making, to want to be included in their own care [21]. In a recent systematic review, older adolescent age was associated with greater responsibility for the decision to lose weight, the weight loss approach, and food choices; for each additional year of age, adolescents perceived less parental involvement in their weight loss [27]. It follows that regardless of any direct changes, adolescents’ inclusion in their own care is of intrinsic value. Indeed, there are data to suggest the value in involving adolescents in the decision-making process to set goals based on their preferences, priorities, and circumstances [21]. Specifically, in a previous study by Kebbe and colleagues (2019), HCPs actively involved adolescents in their own care, and often provided confidential care (i.e.*,* private consultations with adolescents excluding parents) to develop mutual trust and rapport and identify their unique preferences [21]. In further addressing role responsibilities and family dynamics in managing weight, CPGs suggest the health care team probe for and diagnose unhealthy communication patterns with families [10]. HCPs in our study echoed this recommendation as they observed how some parents held adolescents accountable for change, either due to adolescents’ increasing autonomy or parents’ own mental health. This further highlights the importance of considering the family as a unit when offering health services for managing obesity.

HCPs in our study emphasized the importance of reducing weight bias and stigma on a social and clinical level due to the impact it may have on adolescents’ efforts to change lifestyle behaviors. They described the need for a “universal messaging system” for adolescents with obesity, where their lifestyle habits are not subject to scrutiny because of their weight status. Blame and shame can prevent adolescents and their parents from seeking health services and worsen obesity by creating additional barriers to lifestyle behavior change, such as increased binge eating and decreased exercise and physical activity [28]. Perceptions and experiences of bias and stigma can de-motivate individuals from adopting healthy lifestyles [29]. These data highlight the role that HCPs can play by modelling non-biased practices and behaviors, including using people-first language, helping families to understand current and future health risks, ensuring a safe and welcoming clinic environment, and attending to a range of domains – cognitive, affective, behavioral, and social – in their work with adolescents [28]. In our study, HCPs particularly discussed the importance of promoting positive self-image and acceptance in adolescents to help alleviate weight bias concerns; this practice may be considered alongside CPG recommendations to support rearing patterns by parents to help improve adolescent’s self-esteem and potential dissatisfaction with body image [10].

Our study has some limitations to acknowledge. For example, most participating HCPs were female and Caucasian, so some of our findings may be less applicable to samples with other descriptive characteristics. Further, while we strived for data saturation via concurrent data collection and analysis, there is a possibility that HCPs in our sample inadvertently omitted to identify some of their practices due to the wide scope of questioning. Finally, group dynamics may have been influenced by competition for dominance and potential conformity to answers. To address these issues and enhance meaning in our focus groups, moderators leading the focus groups had experience in leading efficient discussions, were conscious of self-HCP and HCP-to-HCP interactions and inclusive of all members, conducted multiple focus groups from multiple sites, and ensured internal and external homogeneity in data analysis.

## Conclusions

HCPs in our study described their practices in delivering health services for weight management to adolescents with obesity, which covered weight loss, treatment expectations, family-centered care, tailored interventions, and weight bias and stigma. While all practices aligned with recommendations from recent CPGs, HCPs offered further insight regarding *what* and *how* they offered care to adolescents with obesity. These findings highlight the range of practices that can be applicable to adolescent weight management, and the importance of identifying which practices are most effective. These findings may help to inform research questions and recommendations when CPGs are updated in the future.

## Supplementary information

**Additional file 1: Table 1.** Consolidated criteria for reporting qualitative studies (COREQ). Reporting checklist for qualitative research

**Additional file 2: Table 2.** Interview guide exploring health care providers’ delivery of weight management health services to adolescents with obesity.

## Data Availability

“The datasets used and/or analysed during the current study are available from the corresponding author on reasonable request.”

## Abbreviations

CHAL: Centre for Healthy Active Living
CPGs: Clinical practice guidelines
HCPs: Health care providers
PCWH: Pediatric Centre for Weight and Health
QD: Qualitative description
