# Supplementary material for: Health care providers’ weight management practices for adolescent obesity and alignment with clinical practice guidelines: a multi-centre, qualitative study
Source: BMC Health Serv Res. 2020 Sep 10;20:850. doi: 10.1186/s12913-020-05702-8 (PMC7488259; doi:10.1186/s12913-020-05702-8)
Supplement: Supplementary file 2 — Additional file 2: Table 2. Interview guide exploring health care providers’ delivery of weight management health services to adolescents with obesity. [file 12913_2020_5702_MOESM2_ESM.docx]

**Table 2.** Interview guide exploring health care providers’ delivery of weight management health services to adolescents with obesity.

1. Describe your role as a health care provider in a multidisciplinary clinical care setting for pediatric weight management.
2. What are some general observations you can make about teens who seek weight management support?
3. What factors do you consider in providing support to teens with obesity who attend the clinic?
4. What strategies do you use in providing support to teens with obesity who attend the clinic?
5. What works? What doesn’t work?
6. Any other comments?
